# Multi-view object pose distribution tracking for pre-grasp planning on mobile robots

**DOI:** 10.3389/frobt.2025.1683931

**Published:** 2026-01-14

**Authors:** Lakshadeep Naik, Thorbjørn Mosekjær Iversen, Jakob Wilm, Norbert Krüger

**Affiliations:** 1 SDU Robotics, Mærsk Mc-Kinney Møller Institute (MMMI), Faculty of Engineering, University of Southern Denmark, Odense, Denmark; 2 Danish Institute for Advanced Studies (DIAS), Odense, Denmark

**Keywords:** pose estimation, uncertainty quantification, pose distribution tracking, multi-camera fusion, mobile manipulation

## Abstract

The ability to track the 6D pose distribution of an object while a mobile manipulator is still approaching it can enable the robot to pre-plan grasps, thereby improving both the time efficiency and robustness of mobile manipulation. However, tracking a 6D object pose distribution on approach can be challenging due to the limited view of the robot camera. In this study, we present a particle filter-based multi-view 6D pose distribution tracking framework that compensates for the limited view of the moving robot camera while it approaches the object by fusing observations from external stationary cameras in the environment. We extend the single-view pose distribution tracking framework (PoseRBPF) to fuse observations from external cameras. We model the object pose posterior as a multi-modal distribution and introduce techniques for fusion, re-sampling, and pose estimation from the tracked distribution to effectively handle noisy and conflicting observations from different cameras. To evaluate our framework, we also contribute a real-world benchmark dataset. Our experiments demonstrate that the proposed framework yields a more accurate quantification of object pose and associated uncertainty than previous research. Finally, we apply our framework for pre-grasp planning on mobile robots, demonstrating its practical utility.

## 1 Introduction

Inspired by the success of mobile logistic robots in warehouses, mobile manipulation (MM) is also making strides toward solving various day-to-day tasks, like assisting cafeteria staff in cleaning cafeteria tables (Garcia-Haro et al., 2020) and handling and transporting supermarket products (Zia Ur Rahman et al., 2022). The success of such robots largely depends on two factors: *time efficiency* and *robustness* (Hvilshøj et al., 2012; Thakar et al., 2023; Sereinig et al., 2020). For example, in the case of a cafeteria cleaning robot, tables must be cleaned quickly so that they can be made available for new customers. Failure to grasp can have serious consequences, such as spilling leftover coffee from a mug, damaging the object, or even disturbing other objects in the vicinity, thereby making it difficult for the robot to complete the task.

Pre-grasp planning can improve both the *time efficiency* and *robustness* of the mobile manipulation (Naik, 2024). For example, i) by determining the optimal base poses for grasping objects (Naik et al., 2024b; Naik et al., 2024a; Jauhri et al., 2022a; Makhal and Goins, 2018; Vahrenkamp et al., 2013), grasping time can be optimized, or ii) by evaluating whether it is safe to attempt a grasp, grasping failures can be reduced (Naik et al., 2025; Shi et al., 2021). Like grasp planning for rigid objects, pre-grasp planning often requires the 6D pose estimate of the objects. Pose estimates are usually obtained using the robot’s onboard camera; if the robot can track the object poses while navigating to the manipulation scene, it can already start pre-grasp planning. However, it is not always possible to have a good view of the objects while approaching, which can make it difficult to track the poses.

Modern indoor environments are often equipped with cameras for various purposes, such as occupancy tracking, surveillance, smart office solutions, and more. These cameras often have an unoccluded overview of the objects. However, due to their greater distance from the objects, they also capture limited pixel information, making it difficult to rely solely on them for accurate 6D pose estimation. Furthermore, for pre-grasp and grasp planning, poses are required in the robot base frame. Transforming poses estimated in the external camera frames to the robot base frame introduces additional uncertainty due to the inherent uncertainties associated with mobile base localization. Nevertheless, these external cameras have the potential to enable better 6D object pose tracking when used in combination with the robot camera.

**FIGURE 1:**
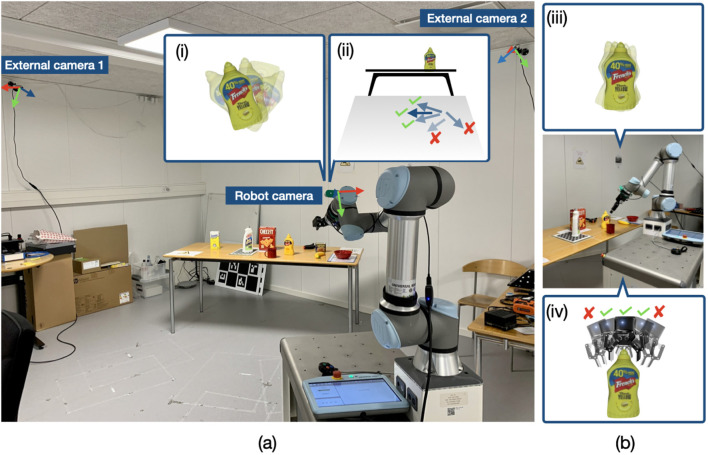
Multi-camera setup for pre-grasp planning. **(a)** Determining whether the robot should navigate to the selected base pose for grasping. **(b)** Determining whether the robot should attempt grasp using the selected grasp pose. a-i) Object pose uncertainty from distance. a-ii) Object pose uncertainty projected onto the base pose space. b-iii) Object pose uncertainty after navigating to the base pose for grasping. b-iv) Object pose uncertainty projected onto the grasp pose space.

Relying on a single best guess of the object pose is always risky, as the accuracy of estimated object poses is influenced by factors such as occlusions, object symmetry, self-occlusions, and lighting conditions (Xiang et al., 2017; Tremblay et al., 2018; Shi et al., 2021). This risk can be reduced by also maintaining and exploiting the uncertainty in the estimate, which is usually expressed as a 6D object pose distribution (Manhardt et al., 2019; Deng et al., 2021). For example, uncertainty can be used to assess the probability of the availability of inverse kinematics (IK) solutions at the estimated base pose for grasping by projecting the pose distribution onto the base pose space (Figures 1a-i and ii). Similarly, uncertainty can be utilized to evaluate the probability of a successful grasp by mapping the pose distribution onto the grasp pose space (Figure 1b-iii and iv).

We propose a multi-view 6D object pose distribution tracking framework that, in addition to the robot camera views, also fuses views from the external cameras in the environment for pose distribution tracking. Specifically, we extend the PoseRBPF (Rao–Blackwellized Particle Filter) (Deng et al., 2021) to utilize information from the external cameras.

Ideally, fusing observations from external cameras should improve the accuracy of the estimated pose distributions. However, multi-camera fusion also introduces several challenges. For example, how do we ensure that during fusion, good observations from some cameras are not corrupted by poor observations from others? and, how do we determine which observations to trust when cameras have contradictory observations? Moreover, a single object pose is required for robotics tasks. However, deriving a single pose from multimodal pose distributions is not trivial. Finally, there are no benchmarks for evaluation that reflect a multi-camera setup for pre-grasp planning (Figure 1a), where the external cameras are positioned far away from the objects while the robot camera views are expected to improve as the robot moves closer to the objects.

We extend the multi-view object pose distribution tracking framework for pre-grasp planning initially introduced in Naik et al. (2022) to address these challenges. In Naik et al. (2022), multi-view fusion was performed by taking the product of observation likelihoods from all camera views without considering potentially unreliable observations from certain cameras. In addition, the standard re-sampling and pose estimation techniques proposed in Deng et al. (2021) were adopted, which did not consider the additional complexities introduced by the multi-camera setup for pre-grasp approach in mobile manipulation. Finally, the framework was evaluated only on a simulated dataset without any real-world evaluation. In this study, we address these limitations through the following contributions.

- To effectively deal with poor observations and conflicting evidence, we propose:
  1. *multi-view exclusion*—a failure model to determine which camera views should be considered during fusion;
  2. *adaptive re-sampling*—a re-sampling strategy to avoid premature convergence to a particular hypothesis before sufficient evidence is obtained from all camera observations;
  3. *pose estimation from tracked distribution* that maintains multiple pose hypotheses when sufficient temporal and spatial evidence is not available to confidently converge on a single pose estimate;
- *Real-world benchmark dataset and evaluation*. We contribute a novel “multi-view YCB object pose tracking dataset for mobile manipulation (MY-MM)” and conduct an extensive evaluation on this real-world dataset.
- *Application*. We exemplify the importance of maintaining object pose uncertainties during pre-grasp planning on mobile robots for the tasks shown in Figure 1, evaluating:
  1. the availability of valid IK solutions for grasping the object using the estimate of the selected base pose;
  2. grasp success using the estimate of the selected grasp pose.

### 1.1 Related research

In this section, we summarize related research in 6D object pose estimation, pose distribution estimation, and multi-view pose estimation, and then we discuss our contribution.

#### 1.1.1 6D object pose estimation

Before the advances in deep learning (Krizhevsky et al., 2012), 6D object pose estimation was often addressed using template-based (Choi and Christensen, 2012b; Lim et al., 2014; Hinterstoisser et al., 2011) or feature-based methods (Drost et al., 2010; Choi and Christensen, 2012a; Collet et al., 2011). In the past decade, research focus has shifted to using data-driven learning-based approaches. Initial work focused on end-to-end regression from image to 6D pose (Kendall et al., 2015). Later studies have tried to better deal with object symmetry and occlusions using novel architectures, loss-function (Xiang et al., 2017), solving orientation as classification (Kehl et al., 2017; Corona et al., 2018), or using keypoints (Pavlakos et al., 2017; Rad and Lepetit, 2017; Peng et al., 2019; Haugaard and Buch, 2022). Furthermore, since robotics tasks such as grasping require very high accuracy, iterative methods have been also developed (Li Y. et al., 2018; Manhardt et al., 2018; Wang et al., 2019; Zakharov et al., 2019) for refining the 6D object poses. However, all these methods only provide best-guess pose estimates without any information regarding associated uncertainty.

#### 1.1.2 Object pose distribution estimation

Pose uncertainties are traditionally modeled using unimodal distributions, such as Gaussians. However, given that pose distributions are often multimodal, it is preferable to employ multimodal distributions, such as a mixture of Gaussians or histograms.

Most existing research has focused solely on estimating rotational distribution (Okorn et al., 2020; Murphy et al., 2021; Iversen et al., 2022), assuming that good translation estimates are available. Research involving the estimation of a full 6D object pose distribution typically either allow multiple pose predictions from the network (Manhardt et al., 2019) or separately model translation and rotational distributions (Glover et al., 2012; Deng et al., 2019; Deng et al., 2021). Translational uncertainty is usually modeled by a simple Gaussian, while rotational uncertainty is modeled using a Bingham mixture model (Glover et al., 2012; Okorn et al., 2020) or a multi-modal histogram distribution (Corona et al., 2018; Deng et al., 2019).

Moreover, most such studies do not directly predict the object pose. They infer the pose either by comparing the object image against the rendered images of the object (Corona et al., 2018), using a learned comparison function against image dictionaries (Okorn et al., 2020), or by codebook matching and training a de-noising auto-encoder for generating codes (Sundermeyer et al., 2018; Deng et al., 2021). Others have represented the distributions implicitly, using a neural network that estimates the probability of a candidate pose given the input image (Murphy et al., 2021; Haugaard et al., 2023).

In this study, we separately model translational and rotational distributions and use codebook matching to determine the likelihood of different rotations.

#### 1.1.3 Multi-view object pose estimation

Multi-view pose estimation involves estimating the 6D pose of an object by considering multiple views of it (Collet and Srinivasa, 2010; Susanto et al., 2012; Kanezaki et al., 2018; Li C. et al., 2018; Xie et al., 2019; Labbé et al., 2020). Sequentially integrating additional views of an object over time for pose estimation is referred to as “object pose tracking.” This includes object-based SLAM methods (Salas-Moreno et al., 2013; Mendes et al., 2016) or active vision methods (Zeng et al., 2017; Kaba et al., 2017; Deng et al., 2020; An et al., 2024) that capture multiple views of an object and improve the pose estimate over time. Some recent research has also concentrated on 6D object pose tracking in a manipulation context (Deng et al., 2019; Wang et al., 2020; Wen et al., 2020).

Other approaches concentrate on simultaneously using multiple views of an object from various camera viewpoints. Some of these methods jointly estimate the 3D pose from multiple object views (Helmer et al., 2010; Su et al., 2015; Kanezaki et al., 2018), others first generate pose estimates in each view and then employ various techniques to fuse multiple pose estimates into one (Collet and Srinivasa, 2010; Susanto et al., 2012; Li C. et al., 2018; Xie et al., 2019; Labbé et al., 2020), while recent work (Haugaard and Iversen, 2023) has used learned 2D–3D correspondence distributions for multi-view pose estimation.

However, not much prior research exists in multi-view full-pose distribution estimation. Erkent et al. (2016) have introduced an approach for integrating the 6D pose estimates from different views by assuming Gaussian uncertainty in each view. As discussed above, a unimodal distribution is not sufficient for modeling symmetric or occluded objects. They also assume only stationary cameras and do not incorporate any motion model for tracking the estimated pose distribution, which is essential for manipulation tasks. To our knowledge, there is no existing research that explores multiple simultaneous views along with sequential temporal integration to estimate multimodal object pose distributions.

## 2 Materials and methods

### 2.1 Problem definition

We define the problem as 6D object pose distribution tracking using the robot’s onboard camera and external cameras in the environment for pre-grasp planning on mobile robots. Let $r_{t}$ , $e_{n,t}$ , $o_{t}$ , and $w_{t}$ be the robot camera, external camera n∈[1 …N−1] , object, and world frame at time t , respectively. $T_{b}^{a}\in SE\left(3\right)$ refers to the transformation from frame a to b . Then, given:

- the transformation of each camera w.r.t the world frame $\left(T_{r_{t}}^{w_{t}},T_{e_{1,t}}^{w_{t}},\ldots ,T_{e_{\left(N-1\right),t}}^{w_{t}}\right)$ ,
- the motion of the robot camera $\left(T_{r_{t}}^{r_{t-1}}\right)$ ,
- a textured CAD model of the object,
- an initial (at t=0 ) object detection (2D bounding box),
- observations (RGB-D images) from external cameras (Zte1,Zte2,…,Zte(N−1)) and the robot camera (Ztr) at time t .

Our objective is to estimate and track the object pose distribution $P\left(X_{t}\right)$ (where $X_{t}\in SE\left(3\right)$ ) by fusing observations from external cameras Zten in addition to the robot camera Ztr and subsequently infer pose estimate ${\hat{X}}_{t}$ from the tracked distribution.

Our approach is inspired by the single-camera 6D pose distribution tracking framework PoseRBPF (Deng et al., 2021). We extend PoseRBPF to the pre-grasp planning multi-camera setup (Figure 1a). In the subsequent sections, we briefly introduce PoseRBPF, including relevant mathematical details for multi-view extensions (Section 2.2), and then present the proposed multi-view extensions (Section 2.3).

### 2.2 PoseRBPF

The posterior distribution of the 6D object pose given the observations up to time t can be expressed as $P\left(X_{t}|Z_{1:t}\right)$ , where $X_{t}$ is a 6D state variable and $Z_{1:t}$ refers to observations from time 1 to t . The 6D state variable $X_{t}$ consists of rotational $R_{t}$ and translational $T_{t}$ components. Since dense sampling in SE(3) is computationally intractable, PoseRBPF models the rotational component $R_{t}$ as conditioned on the translational component $T_{t}$ as described in Equation 1.

$$P\left(X_{t}|Z_{1:t}\right)=P\left(T_{t},R_{t}|Z_{1:t}\right)=P\left(T_{t}|Z_{1:t}\right)P\left(R_{t}|T_{t},Z_{1:t}\right)$$ (1)

In addition, particles are sampled only over the translation space marginalized over the rotation (Rao–Blackwellization) (Murphy and Russell, 2001; Doucet et al., 2001). This approximation allows efficient sampling in the 6D space.

To effectively capture the object’s symmetry and visual ambiguity arising from occlusions, the rotation conditioned on the translation $P\left(R_{t}|T_{t},Z_{1:t}\right)$ is modeled as a 3D histogram distribution. Each bin in the histogram $r_{i,j,k,t}\in SO\left(3\right)$ represents the discretized rotational component of the object pose at time t , with i , j , and k denoting the elevation, azimuth, and bank indices, respectively. Discretization is performed with a resolution of 5°. As a result, the histogram distribution consists of 37 × 72 × 72 (elevation, azimuth, bank) bins. The probability of $r_{i,j,k,t}$ is $P\left(r_{i,j,k,t}|T_{t},Z_{1:t}\right)$ , where i∈[1.37] , j∈[1.72] , and k∈[1.72] .

Thus, the m th particle $X_{t}^{m}$ at time t is represented by a translation $T_{t}^{m}$ and a discretized rotation distribution conditioned over the translation $P\left(R_{t}^{m}|T_{t}^{m},1:t\right)$ :

$$X_{t}^{m}=\left\{T_{t}^{m},P\left(R_{t}^{m}|T_{t}^{m},Z_{1:t}\right)\right\}.$$ (2)

The posterior distribution at time t is approximated by a set of M particles $X_{t}$ expressed as

$$X_{t}={\left\{X_{t}^{m}\right\}}_{m=1}^{M}.$$ (3)

During initialization, the translational component $T_{t}^{m}$ of the particles are sampled around the available translation estimate of the object, assuming Gaussian uncertainty. The discretized rotation distribution conditional on the translation $P\left(R_{t}^{m}|T_{t}^{m},1:t\right)$ for each particle is initialized as a uniform distribution.

At each time-step t , the set of particles from the previous time-step $X_{t-1}$ are propagated to the current time-step using a motion model. The motion propagation involves sequentially applying transformations: $T_{r_{t-1}}^{r_{t}}$ (inverse of the motion of the robot camera frame), $T_{o_{t-1}}^{r_{t-1}}$ (transformation from the previous robot camera frame to the previous object frame), and $T_{o_{t}}^{o_{t-1}}$ (motion of the object frame).

$$T_{o_{t}}^{r_{t}}=T_{r_{t-1}}^{r_{t}}\cdot T_{o_{t-1}}^{r_{t-1}}\cdot T_{o_{t}}^{o_{t-1}}.$$ (4)

The observation likelihood $P\left(Z_{t}|T_{t},R_{t}\right)$ is measured using the degree of agreement between observation $Z_{t}$ and the propagated particles $X_{t}$ . A codebook of vector embeddings is pre-computed for the discretized rotation space of the object using a de-noising auto-encoder (Sundermeyer et al., 2018). The pre-computed encodings from the codebook for different rotations of the object are then compared with the encodings obtained from the current observation $Z_{t}$ .

As each particle $X_{t}^{m}$ consists of a translation $T_{t}^{m}$ and a discretized rotation distribution conditioned on the translation $P\left(R_{t}^{m}|T_{t}^{m},1:t\right)$ (Equation 2), the observation likelihood $P\left(Z_{t}|T_{t}^{m},R_{t}^{m}\right)$ for each particle m is a histogram, with each bin containing the likelihood of the discretized rotation.

The importance factor (weight) of an individual particle is directly proportional to its observation likelihood (Thrun, 2002).

$$w_{t}^{m}\propto P\left(Z_{t}|X_{t}^{m}\right).$$

The weight $w_{t}^{m}$ for each particle $X_{t}^{m}$ is calculated by marginalizing over the observation likelihood for rotation samples, as shown in Equation 5:

$$\begin{array}{cc} w_{t}^{m} & \propto \int P\left(Z_{t}|T_{t}^{m},R_{t}^{m}\right)\cdot P\left(R_{t}^{m}\right)\;dR\\ & \approx \underset{i,j,k}{\sum }P\left(Z_{t}|T_{t}^{m},r_{i,j,k,t}^{m}\right)\cdot P\left(r_{i,j,k,t}^{m}\right). \end{array}$$ (5)

Re-sampling involves increasing the number of relevant particles—that is, the particles with higher weights are more likely to be duplicated, and those with lower weights are more likely to be discarded (Thrun, 2002). After the re-sampling step, a multi-set of important particles $X_{t}$ is obtained.

The translational component $T_{t}^{m}$ of these particles represents the posterior translation distribution $P\left(T_{t}|Z_{1:t}\right)$ at time t . To obtain the posterior rotation distribution $P\left(R_{t}|Z_{1:t}\right)$ , the individual observation likelihoods $P\left(Z_{t}|T_{t}^{m},R_{t}^{m}\right)$ of each particle m are marginalized. The posterior rotational distribution $P\left(R_{t}|Z_{1:t}\right)$ with temporal fusion up to time t is obtained using Bayes’ rule.

Finally, the 6D pose estimate of the object from the distribution is computed. The translation estimate ${\hat{T}}_{t}$ of the object pose ${\hat{X}}_{t}$ at time t is obtained by taking an average of the posterior translation distribution. The rotation estimate ${\hat{R}}_{t}$ is obtained by taking the weighted average of the samples in the neighborhood of a rotation estimate at the previous time step ${\hat{R}}_{t-1}$ after applying the motion from times t−1 to t .

### 2.3 Proposed approach

**FIGURE 2:**
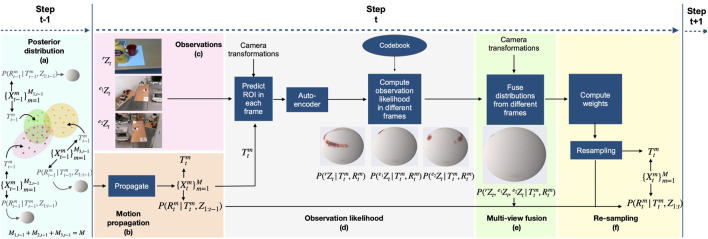
Rao–Blackwellized particle filter proposed by Deng et al. (2021) extended for multi-view 6D pose distribution tracking.

In this section, we first provide a brief overview of the proposed multi-view pose distribution tracking framework using Figure 2 and then present our contributions in detail.

#### 2.3.1 Overview

Figure 2 describes the architecture of the proposed multi-view 6D object pose distribution tracking framework for a three-camera setup comprising a robot camera and two external cameras. The posterior distribution is modeled as a multi-modal distribution (Section 2.3.2). The particles approximating the posterior distribution at time t−1 (Figure 2a) are propagated to time t using the motion model (Figure 2b; Section 2.3.3). Observation likelihoods for these propagated particles are then computed for each camera (Figures 2c,d), as proposed in PoseRBPF. Next, the observations from different cameras are fused (Figure 2e; Section 2.3.4). Each particle is then weighed based on the fused observations, followed by adaptive re-sampling (Section 2.3.5). The posterior distributions at time t are obtained using temporal fusion. The 6D pose is then estimated using the pose distribution obtained (Section 2.3.6), and the re-sampled particles are advanced to the next time-step.

It should be noted that the robot camera frame $r_{t}$ is used as a reference frame, and both object pose distribution $P\left(X_{t}\right)$ and pose estimate ${\hat{X}}_{t}$ are estimated in this frame.

#### 2.3.2 Multi-modal posterior distribution

As each camera can have different translation and rotation beliefs, we model both translation and rotation posterior distributions as multi-modal distributions. We assume that the uncertainty in the translation $T_{t}$ follows a Gaussian distribution in each camera. By combining the Gaussian distributions from all camera frames, the resultant posterior translation distribution is modeled as a mixture of Gaussians, as shown in Equation 6:

$$P\left(T_{t}|Z_{1:t}\right)∼\overset{N}{\underset{n=1}{\sum }}\;\phi_{n}\;N\left(T_{t};\;\mu_{n,t},\Sigma_{n,t}\right),$$ (6)

where N is the number of camera frames, N is the normal distribution with the parameters $\mu_{n,t}$ (mean) and $\Sigma_{n,t}$ (co-variance matrix), and $\phi_{n}$ is a weight for the particular camera.

During the initialization of the tracking (at t=0 ), the translation component $T_{t}^{m}$ of the particles are sampled around the translation estimate $\mu_{n,0}$ in each camera n obtained either from an object detector (Wu et al., 2019) or an alternate pose estimator (Xiang et al., 2017; Wang et al., 2019). In the case of an object detector, the bounding box center and corresponding depth (in the camera depth image) is used as a translation estimate. The particles are then sampled around these estimates assuming Gaussian noise with standard deviation $\Sigma_{n,0}$ in each camera n . If more than one camera provides an initial translational estimate $\mu_{n,0}$ , then the resultant posterior translation distribution is a mixture of Gaussians. For the three-camera setup in Figure 2, the posterior translation distribution is represented using three Gaussians (Figure 2a). The discretized rotation distribution conditional on the translation $P\left(R_{t}^{m}|T_{t}^{m},1:t\right)$ for each particle is initialized as a uniform distribution similar to PoseRBPF.

As the camera from which a particle $X_{t}^{m}$ originated at t=0 is required during the re-sampling step (Section 2.3.5), we represent the particles in $X_{t}$ (Equation 3) originating from camera n using the set $X_{t}^{n}$ . For N cameras, there exists N such particle sets $X_{t}^{n}$ , where each $X_{t}^{n}$ is a subset of $X_{t}$ $\left(X_{t}^{n}\subseteq X_{t}\right)$ with size $|X_{t}^{n}|=M_{n,t}$ and $X_{t}^{i}\cap X_{t}^{j}=\emptyset$ . The total number of particles M in particle set $X_{t}$ is kept constant, so that at any time t , $M=M_{1,t}+M_{2,t}+\cdots \;.+M_{N,t}$ . This is ensured during the re-sampling step.

#### 2.3.3 Motion propagation

The particles originating from the robot camera frame are propagated as described in Equation 4 (same as PoseRBPF). The motion propagation for particles originating from the external camera frames (as described in Equation 7) involves sequentially applying transformations: $T_{e_{n,t}}^{r_{t}}$ (transformation from the current robot camera frame to the current external camera frame), $T_{e_{n,t-1}}^{e_{n,t}}$ (inverse of the motion of the external camera frame), $T_{r_{t-1}}^{e_{n,t-1}}$ (transformation from the previous external camera frame to the previous robot camera frame), $T_{o_{t-1}}^{r_{t-1}}$ (transformation from the previous robot camera frame to the previous object frame), and $T_{o_{t}}^{o_{t-1}}$ (motion of the object frame):

$$T_{o_{t}}^{r_{t}}=T_{e_{n,t}}^{r_{t}}\cdot T_{e_{n,t-1}}^{e_{n,t}}\cdot T_{r_{t-1}}^{e_{n,t-1}}\cdot T_{o_{t-1}}^{r_{t-1}}\cdot T_{o_{t}}^{o_{t-1}}.$$ (7)

External cameras and objects are assumed stationary, and hence their motion from times t−1 to t is 0 (i.e., $T_{e_{n,t-1}}^{e_{n,t}}$ and $T_{o_{t}}^{o_{t-1}}$ are identity matrices). The uncertainty of the transformation from the external to the robot camera $T_{e_{n,t}}^{r_{t}}$ is modeled as Gaussian noise. The uncertainty of the relative motion of the robot camera $\left(T_{r_{t-1}}^{r_{t}}\right)$ is modeled as Gaussian noise with dispersion proportional to the relative motion (Thrun, 2002).

At the end of the motion propagation step, a new particle set $X_{t}$ is obtained, which contains the updated particles from the previous time step $X_{t-1}$ after applying the motion. Each particle $X_{t-1}^{m}$ in $X_{t-1}$ with its translation $T_{t-1}^{m}$ and rotation distribution conditioned on the translational component $P\left(R_{t-1}^{m}|T_{t-1}^{m},Z_{1:t-1}\right)$ is propagated from times t−1 to t , resulting in $T_{t}^{m}$ and $P\left(R_{t}^{m}|T_{t}^{m},Z_{1:t-1}\right)$ .

NOTE: In the remaining sections, frames are represented by integers ranging from 1 to N for simplification. Specifically, the robot camera frame r is denoted by 1, while the external camera frames $e_{2},\ldots ,e_{N}$ are represented by integers from 2 to N . Additionally, when a translation or rotation variable is not explicitly associated with a specific camera frame, it should be assumed to be in the robot camera frame, such that both $T_{t}^{m}$ ${\text{and}\;}^{1}T_{t}^{m}$ represent the translation estimate in the robot camera frame r .

#### 2.3.4 Multi-view fusion

As the observation likelihoods $P\left({\;\;}^{n}\;Z_{t}|T_{t}^{m},R_{t}^{m}\right)$ ^1^ obtained for each particle m in camera n are assumed to be conditionally independent of each other, the joint observation likelihood can be obtained by fusing the individual observation likelihoods (Durrant-Whyte and Henderson, 2016) as described in Equation 8:

P 1Zt,.,ZtN|Ttm,Rtm=∏n=1NPZtn|Ttm,Rtm. (8)

The observation likelihoods from different cameras are first transformed into a common reference frame. The likelihoods of corresponding bins across views are then fused to obtain the resultant likelihood. While this ensures consistency across views and mitigates potential scale bias, such a fusion is unable to handle erroneous observations (Khaleghi et al., 2013). For instance, if the object is not visible in one of the cameras (i.e., empty histogram), the fused observation likelihood will also be an empty histogram.

To address this issue, additive/Laplace smoothing (Schütze et al., 2008) can be performed on the individual observation likelihoods before the fusion. However, if camera n has a poor view of the object (for example, due to occlusions), the observation likelihood $P\left({\;\;}^{n}\;Z_{t}|T_{t}^{m},R_{t}^{m}\right)$ obtained for particle m in camera frame n will most likely be inaccurate. When combined with good observations from other cameras, such an observation can corrupt the overall fusion result.

We address this by defining a failure model to assess whether an observation from a particular camera should be included in the fusion process (Khaleghi et al., 2013). Let $\gamma_{n,t}$ represent the value of maximum likelihood for the observation Ztn over all possible translations and rotations in the particle set.

γn,t=maxm∈1.M,i∈1.37,j∈1.72,k∈1.72PZtn| nTtm, nRtm= nri,j,k,tm. (9)

It is shown in Deng et al. (2021) that $\gamma_{n,t}$ is correlated with the degree of object occlusion. If $\gamma_{n,t}$ falls below 0.6, which corresponds to approximately 65% of the object being occluded, then the object can no longer be reliably tracked. We use this insight to define the failure model to determine whether observation likelihoods for particles in camera n (Figure 2d) should be included in the fusion (Figure 2e). The observation likelihood $P\left({\;\;}^{n}\;Z_{t}|T_{t}^{m},R_{t}^{m}\right)$ for particle m in camera frame n is included during fusion only if it is $\gamma_{n,t}$ greater than pre-defined threshold value $\gamma_{\text{thres}}$ .

Observation likelihoods in different frames are fused together (Algorithm 1). Prior to fusion, observation likelihoods from different frames are transformed into a robot camera frame, and additive/Laplace smoothing is applied by adding a small minimum likelihood $\epsilon_{\text{fus}}$ to all rotations in the discretized rotation space. The observation likelihood from frame n is included in the fusion if $\gamma_{n,t}$ exceeds $\gamma_{\text{thres}}$ .

Algorithm 1 **Multi-view fusion.**

1: input: {P(Ztn|nTtm, nRtm)}n=1n=N,{γi,t}1N

2: output: P( 1Zt,.,ZtN|Ttm,Rtm)

3:
4: P( 1ZN,.,ZtN|Ttm,Rtm)=1

5: **for** i in range(1,N) **do**
6:  **if** $\gamma_{i,t}>\gamma_{\text{thres}}$ **then**
7:   transform P(Zti| iTtm, iRtm) to P(Zti|Ttm,Rtm)

8: P( 1Zt,.,ZtN|Ttm,Rtm) ⋅=P(Zti|Ttm,Rtm)+ϵfus

9:  **end if**
10: **end for**
11: return P( 1Zt,.,ZtN|Ttm,Rtm)

At the end of this step, the joint observation likelihood is obtained for each particle in the particle set $X_{t}$ (Figure 2e).

Note that in the rest of this study, for brevity,  1Zt, 2Zt,…,Ztn are represented as $Z_{t}$ .

#### 2.3.5 Adaptive re-sampling

As described in Section 2.3.4, the decision to include observation likelihood $P\left({\;\;}^{n}\;Z_{t}|T_{t}^{m},R_{t}^{m}\right)$ of particle m in camera n during the fusion is based on the $\gamma_{n,t}$ (Equation 9). Moreover, as explained in Section 2.3.2, at t=0 , for each camera n , with the translation estimate of the object $\mu_{n,0}$ , $M_{n,t}$ number of particles are sampled around this translation estimate assuming Gaussian distributed noise. These particles, denoted by the set $X_{0}^{n}$ , represent the observation of camera n at t=0 . Prior to re-sampling at time t , these particles are denoted using the set ${\bar{X}}_{t}^{n}$ . Consequently, before the filter converges, the particles in the set ${\bar{X}}_{t}^{n}$ are more likely to have the maximum likelihood $\gamma_{n,t}$ for the camera’s n th observation Ztn compared to other sets of particles that represent other camera observations.

Conventional re-sampling techniques do not distinguish between particles representing different camera observations. Thus, if camera n has a poor observation at time t (before convergence) due to motion blur, partial occlusion, and so on, particles ${\bar{X}}_{t}^{n}$ representing the observations of camera n will be more affected as they will have smaller weights than other particles. As a result, there is a possibility that these particles may be eliminated during re-sampling.

Consequently, there is an increased possibility that observations Ztn from camera n may not have a sufficiently high $\gamma_{n,t}$ during subsequent time-steps to be included during the fusion, as all the particles representing its observation were eliminated. As a result, the particle filter may prematurely converge to an inaccurate hypothesis by solely considering observations from other cameras and disregarding observations from camera n .

Separately re-sampling the particles in each ${\bar{X}}_{t}^{n}$ in Equation 3 can ensure that all particles from each of the n sets of particles ${\bar{X}}_{t}^{n}$ are retained throughout the tracking process. However, this can lead to a situation where the set of particles ${\bar{X}}_{t}^{n}$ with a very poor initial translation estimate $\mu_{n,0}$ are also retained forever. Such uninformative particles would only waste computational resources without contributing to improved tracking.

To address this issue, we dynamically adjust the size of the particle sets ${\bar{X}}_{t}^{n}$ based on the average aggregate weight of the particles within these sets. We increase the size of the particle sets with the highest average aggregate weight and reduce the size of the other particle sets to maintain a constant number of particles M at time t . Particles are then individually re-sampled from each of these sets. Thus, we prevent the elimination of particles originating from certain cameras due to a few poor observations in that camera; however, if a camera has consistently poor observations, then these particles are eventually eliminated.

In the rest of this section, we provide technical details of our approach. Readers can optionally proceed directly to Section 2.3.6 without these details.

We refer to our approach as “adaptive re-sampling” (Algorithm 2). It takes $X_{t}$ (the particle set obtained at the end of motion propagation step—Section 2.3.3) and W (the set of weights associated with M particles in $X_{t}$ ) as inputs and returns the new particle set $X_{t}$ containing M particles (Lines 1–2). $r_{\text{idx}}$ contains the re-sampled indices of the particles in set $X_{t}$ that are used for creating new particle set $X_{t}$ .

We let $\text{idxs}\left({\bar{X}}_{t}^{n}\right)$ return the indices of the particles in the particle set $X_{t}$ that originated from the camera n observation at t = 0 and $\text{wts}\left({\bar{X}}_{t}^{n}\right)$ return the weights of these particles

$$\text{wts}\left({\bar{X}}_{t}^{n}\right)=\left\{w_{t}^{m}|m\in \text{idxs}\left({\bar{X}}_{t}^{n}\right)\right\}.$$

Furthermore, “bpsi” (best particle set index) is the index of the ${\bar{X}}_{t}^{n}$ with the highest average aggregate particle weights (Line 4), while “bpibps” (best particle index in the best particle set) is the index of the particle with maximum weight in the best particle set ${\bar{X}}_{t}^{\text{bpsi}}$ (Line 5).

The size of each of the n particle sets ${\bar{X}}_{t}^{n}$ is dynamically adjusted based on the average aggregate weight of the particles in these sets (Lines 6–12). The size of the particle sets ${\bar{X}}_{t}^{n}$ with lowly weighted particles is reduced, while the size of the particle set with the highest average aggregate weight of particles is increased. The particle with the maximum weight from ${\bar{X}}_{t}^{\text{bpsi}}$ is duplicated N −1 times. Particles with the lowest weights from other particle sets (“wpipsn”—worst particle index in particle set ${\bar{X}}_{t}^{n}$ ) are eliminated to ensure that M remains constant. Finally, particles from each ${\bar{X}}_{t}^{n}$ are separately re-sampled (Lines 13–18). This incremental process of replicating the “best” particle followed by re-sampling at each time-step prevents sudden particle impoverishment and helps maintain multiple hypotheses, thereby reducing the risk of mode collapse.

Algorithm 2 **Adaptive re-sampling.**

1: $input:\;{\bar{X}}_{t},W$

2: $output:\;X_{t}$

3: $r_{\text{idx}}=\left[\right]$ ⊳ Multi-set (can contain duplicates)
4: bpsi = ${argmax}_{n}{\left\{\left(\sum \text{wts}\left({\bar{X}}_{t}^{n}\right)\right)/|{\bar{X}}_{t}^{n}|\right\}}_{n=1}^{N}$

5: bpibps = $\text{idxs}\left({\bar{X}}_{t}^{\text{bpsi}}\right)\left[argmax\left\{\text{wts}\left({\bar{X}}_{t}^{\text{bpsi}}\right)\right]\right\}$

6: **for** n∈range(1,N) **do**
7:  **if** n not equal to bpsi **then**
8: $\text{wpipsn}=\text{idxs}\left({\bar{X}}_{t}^{\text{bpsi}}\right)\left[\mathrm{arg min}\left\{\text{wts}\left({\bar{X}}_{t}^{n}\right)\right\}\right]$

9: $\text{idxs}\left({\bar{X}}_{t}^{n}\right)\leftarrow \left(\text{idxs}\left({\bar{X}}_{t}^{n}\right)\backslash \text{wpipsn}\right)$

10: $\text{idxs}\left({\bar{X}}_{t}^{\text{bpsi}}\right)\leftarrow \text{idxs}\left({\bar{X}}_{t}^{\text{bpsi}}\right)\cup \left\{\text{bpibps}\right\}$

11:  **end if**
12: **end for**         ⊳ Re-sampling
13: **for** n∈range(1,N) **do**
14:  **for** $m\in \text{range}\left(1,|{\bar{X}}_{t}^{n}|\right)$ **do**
15:   draw j from $\text{idxs}\left({\bar{X}}_{t}^{n}\right)$ with prob. $\propto \text{wts}\left({\bar{X}}_{t}^{n}\right)$

16: $r_{\text{idx}}\leftarrow r_{\text{idx}}\cup \left[j\right]$

17:  **end for**
18: **end for**
19: $X_{t}=X_{t}\left[r_{\text{idx}}\right]$

20: $return\;X_{t}$

By adopting this strategy, we prevent premature convergence and allow the maintenance of a diverse set of particles for a specific duration so that sufficient temporal evidence is obtained from all cameras before convergence. This duration depends on the number of particles in each of the n particle sets $X_{t}^{n}$ .

#### 2.3.6 Pose estimation from tracked distribution

For the multi-camera setup considered here, initially at t=0 , only uncertain pose estimates are available due to the large distance between the cameras and the objects. Hence, the assumptions such as having a good initial object rotation estimate (at t=0 ) in Deng et al. (2021) are not always valid. To address this, we maintain multiple pose hypotheses when sufficient evidence is not available in the tracked distribution to converge to a single pose estimate. In the following sections, we describe our approach for estimating the translation ${\hat{T}}_{t}$ and rotation ${\hat{R}}_{t}$ components of ${\hat{X}}_{t}$ using the distribution $P\left(X_{t}\right)$ .

##### 2.3.6.1 Translation estimate ${\hat{T}}_{t}$

The posterior translation distribution $P\left(T_{t}|Z_{1:t}\right)$ is modeled as a mixture of Gaussians with M (number of particles) samples. This distribution in general reduces to a unimodal Gaussian distribution as the filter starts to converge. The translation estimate ${\hat{T}}_{t}$ of the object pose ${\hat{X}}_{t}$ at time t is obtained by taking a weighted average of the particles of the dominant Gaussian (i.e., the Gaussian with the highest associated weight) in the posterior translation distribution.

##### 2.3.6.2 Rotation estimate ${\hat{R}}_{t}$

The posterior rotation distribution $P\left(R_{t}|Z_{1:t}\right)$ is modeled as a potentially multimodal histogram distribution. While weighted average can be employed to compute the rotation estimate ${\hat{R}}_{t}$ when the histogram distribution is unimodal, it can yield an incorrect estimate when it is multimodal.

We maintain multiple rotation estimates when sufficient evidence is not present in the posterior rotation distribution $P\left(R_{t}|Z_{1:t}\right)$ to converge to a single pose estimate. Thus, we maintain and track a set of H rotation hypotheses as described in Equation 10,

$$R_{t}=\left\{{\left\{r_{h,t},c_{h,t}\right\}}_{h=1}^{H}\right\}$$ (10)

where $r_{h,t}\in SO\left(3\right)$ is the rotation hypothesis and $c_{h,t}\in R^{1}$ is the associated confidence.

In the subsequent sections, we present our approach. In Section 2.3.6.2, we introduce the notion of *supporting samples* $S_{h,t}$ , and we outline the algorithm for maintaining a set of rotation hypotheses $R_{t}$ in Section 2.3.6.2.

###### 2.3.6.2.1 Supporting samples $S_{h,t}$

Supporting samples $S_{h,t}$ are the discretized rotational components $r_{i,j,k,t}$ in the posterior rotational distribution $P\left(R_{t}|Z_{1:t}\right)$ that can be associated with the rotation hypothesis $r_{h,t-1}\in R_{t-1}$ . A discretized rotational component $r_{i,j,k,t}$ is considered to be part of the *supporting samples* $S_{h,t}$ if it lies in the neighborhood of ${\bar{r}}_{h,t}$ (the rotation hypothesis $r_{h,t-1}$ after applying the camera motion from t −1 to t ) and has a probability greater than $\epsilon_{\text{min}}$ :

$$\begin{array}{c} S_{h,t}=\left\{r_{i,j,k,t}|r_{i,j,k,t}\in P\left(R_{t}|Z_{1:t}\right)\;\wedge \;\right.\\ \left.|r_{i,j,k,t}-{\bar{r}}_{h,t}|\leq \epsilon_{\text{sup}}\;\wedge \;P\left(r_{i,j,k,t}\right)\geq \epsilon_{\text{min}}\right\}, \end{array}$$ (11)

where $\epsilon_{\text{sup}}$ is a threshold angular difference to determine whether the discretized rotational component $r_{i,j,k,t}$ is in the neighborhood of the ${\bar{r}}_{h,t}$ , and $\epsilon_{\text{min}}$ is the minimum probability that a discretized rotational component $r_{i,j,k,t}$ must be part of $S_{h,t}$ .

**FIGURE 3:**
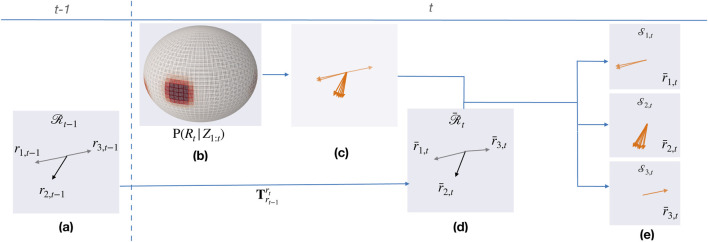
Illustration for determining supporting samples. **(a)** Set of rotation hypothesis at time t−1. **(b)** Posterior rotation distribution $P\left(R_{t}|Z_{1:t}\right)$. **(c)** Rotation samples in $P\left(R_{t}|Z_{1:t}\right)$ with probabilities greater than or equal to $\epsilon_{\text{min}}$. **(d)** Transformed rotation hypothesis from $R_{t-1}$ after applying motion from t−1 to t. **(e)** Supporting samples $S_{h,t}$ at time t for rotation hypothesis $r_{h,t-1}$ in $R_{t-1}$.

Figure 3 illustrates the computation of *supporting samples* for three different rotation hypotheses in a set (Figure 3a). Initially, the posterior rotation distribution $P\left(R_{t}|Z_{1:t}\right)$ is obtained at time t (Figure 3b), and the rotations with probabilities greater than $\epsilon_{\text{min}}$ are extracted (Figure 3c). This is followed by applying the motion from time t−1 to t to all the rotation hypotheses to obtain ${\bar{R}}_{t}$ (Figure 3d). Finally, the *supporting samples* $S_{h,t}$ are obtained for each rotation hypothesis, as in Equation 11 (Figure 3e).

It is assumed that the rotation hypothesis $r_{h,t-1}$ can be associated with the posterior rotation distribution $P\left(R_{t}|Z_{1:t}\right)$ $\left(\text{association}\left(r_{h,t-1},P\left(R_{t}|Z_{1:t}\right)\right)=\text{True}\right)$ if more than $s_{\text{thres}}$ % samples with probabilities greater or equal to $\epsilon_{\text{min}}$ in $P\left(R_{t}|Z_{1:t}\right)$ are in $S_{h,t}$ . For example, in Figure 3, most of the samples with probabilities greater than $\epsilon_{\text{min}}$ in the posterior rotation distribution $P\left(R_{t}|Z_{1:t}\right)$ are in the neighborhood of $r_{2,t-1}$ . Hence, if $s_{\text{thres}}$ is set to 0.8 (80%), only $r_{2,t-1}$ can be associated with the samples in the posterior rotation distribution $P\left(R_{t}|Z_{1:t}\right)$ at time t .

###### 2.3.6.2.2 Maintaining a set of rotation hypotheses

If the samples in the posterior rotation distribution $P\left(R_{t}|Z_{1:t}\right)$ can be associated with the rotation hypothesis $r_{h,t-1}$ , then $r_{h,t-1}$ is updated using the expectation of the supporting samples. However, if the samples in the posterior rotation distribution $P\left(R_{t}|Z_{1:t}\right)$ cannot be reliably associated with the rotation hypothesis $r_{h,t-1}$ , then $r_{h,t-1}$ is split into two hypotheses at time t —one suggested by the data that could be associated with $r_{h,t-1}$ and the other suggested by the data that could not be associated with $r_{h,t-1}$ (Thrun, 2002). The algorithm for maintaining a set of rotation hypotheses is described in Algorithm 3.

Initially, the rotation hypothesis $r_{h,t-1}$ under consideration is updated using the camera motion from times t−1 to t , resulting in ${\bar{r}}_{h,t}$ (Line 6). Subsequently, the supporting samples are determined, and if the rotation hypothesis $r_{h,t-1}$ can be associated with the posterior rotation distribution $P\left(R_{t}|Z_{1:t}\right)$ , $r_{h,t-1}$ is updated by taking the expectation of the supporting samples (Lines 6–10).

If $r_{h,t-1}$ cannot be associated with the $P\left(R_{t}|Z_{1:t}\right)$ , a new rotation hypothesis $r_{h,t}^{\prime }$ is introduced (Lines 12). The confidence in this hypothesis is computed relative to the confidence in the parent hypothesis $r_{h,t-1}$ , similar to multi-hypothesis tracking in Thrun (2002). Finally, the confidences $c_{h,t}$ in all the rotation hypotheses $r_{h,t}$ are normalized (by dividing by the sum of all confidences). Only the rotation hypotheses $r_{h,t}$ with confidence $c_{h,t}$ greater than $c_{\text{thres}}$ are retained for the subsequent time-step (Line 18). The maximum number of hypotheses retained at any time step is given by $1/c_{\text{thres}}$ .

Algorithm 3 **Set of rotation hypotheses.**

1: $input:\;P\left(R_{t}|Z_{1:t}\right),R_{t-1}$

2: $output:\;R_{t}$

3: $P\left(R_{t}\right)\equiv P\left(R_{t}|Z_{1:t}\right)$ ⊳ For simplification purpose
4: $R_{t}=\left\{\right\}$

5: **for** $r_{h,t-1}\in R_{t-1}$ **do**
6: $\text{Compute}\;{\bar{r}}_{h,t}$ ⊳ Using motion from t−1 to t

7: $\text{Compute}\;S_{h,t}$ ⊳ Equation 11
8: $c_{h,t}=P\left(R_{t}={\bar{r}}_{h,t}\right)$ ⊳ Using $P\left(R_{t}\right)$

9:  **if** $\text{association}\;\left(r_{h,t-1},P\left(R_{t}\right)\right)$ **then**
10: $R_{t}=R_{t}\cup \left\{\left\{E\left[r_{i,j,k,t}\in S_{h,t}\right],c_{h,t}\right\}\right\}$

11:  **else**
12: $r_{h,t}^{\prime }=\left\{\begin{array}{cc} E\left[R_{t}\right] & \mathrm{if}P\left(R_{t}\right)\text{is}\text{uni}-\text{modal}\\ {argmax}_{R_{t}}P\left(R_{t}\right) & \text{otherwise} \end{array}\right.$

13: $c_{h,t}^{\prime }=c_{h,t}\cdot P\left(R_{t}=r_{h,t}^{\prime }\right)$

14: $R_{t}=R_{t}\cup \left\{\left\{{\bar{r}}_{h,t},c_{h,t}\right\},\left\{r_{h,t}^{\prime },c_{h,t}^{\prime }\right\}\right\}$

15:  **end if**
16: **end for**
17: $\text{Normalize }c_{h,t}$

18: $return\;\left\{\left\{r_{h,t},c_{h,t}\right\}\in R_{t}|c_{h,t}>c_{\text{thres}}\right\}$

Algorithm 3 thus covers two possible failure cases. The association could not be established between the rotation hypothesis $r_{h,t-1}$ and the posterior rotation distribution $P\left(R_{t}|Z_{1:t}\right)$ because

1.
$P\left(R_{t}|Z_{1:t}\right)$ is a noisy distribution and the rotation hypothesis ${\bar{r}}_{h,t}$ (transformed $r_{h,t-1}$ after applying the camera motion) is a valid estimate;
2.
${\bar{r}}_{h,t}$ (transformed $r_{h,t-1}$ after applying the camera motion) is an invalid estimate but $P\left(R_{t}|Z_{1:t}\right)$ is a valid distribution.

In the first case, the rotation hypothesis $r_{h,t-1}$ remains in the set $R_{t}$ , and confidence in it increases, while confidence in the rotation hypotheses added based on the noisy samples in $P\left(R_{t}|Z_{1:t}\right)$ (that could not be associated with $r_{h,t-1}$ ) decreases as more valid distributions are acquired in subsequent time steps.

In the second case, confidences in the rotation hypotheses added based on the samples in the posterior rotation distribution $P\left(R_{t}|Z_{1:t}\right)$ which could not be associated with hypothesis $r_{h,t-1}$ increases, and simultaneously, confidence in hypothesis $r_{h,t-1}$ decreases as more similar $P\left(R_{t}|Z_{1:t}\right)$ samples are obtained in the subsequent time-steps.

At time t , the rotational hypothesis $r_{h,t}$ with the highest associated confidence $c_{h,t}$ is used as the rotational estimate ${\hat{R}}_{t}$ .

## 3 Results

### 3.1 Experimental evaluation

#### 3.1.1 Experiment objectives

The experiments are designed to verify that the proposed multi-view object pose distribution tracking using a robot and external cameras results in more accurate

- object pose estimation $\hat{X}$ than existing multi-view pose estimation methods;
- uncertainty quantification P(X) than existing single-view uncertainty quantification methods;
- object pose estimation $\hat{X}$ and uncertainty quantification P(X) compared to using individual robot or external cameras alone.

Since our focus is on household objects, we use YCB benchmark (Calli et al., 2015) objects for evaluation. We consider the following baselines.

- PoseRBPF (Deng et al., 2021): a single-camera object pose distribution tracking framework that provides both pose estimates and pose distributions at each time-step.
- CosyPose (Labbé et al., 2020): a multi-camera pose estimation method. It first estimates the object poses in each camera and then matches individual pose hypotheses across different input images to jointly estimate camera viewpoints and 6D object poses in a single consistent scene.

We also conducted three ablation studies to validate our design choices for multi-view fusion, re-sampling, and pose estimation from a tracked distribution. Finally, we present an ablation study to compare the performance with different camera combinations for fusion.

#### 3.1.2 Experiment setup

As the mobile robot approaches the object for grasping using its base and arm motion, the robot camera will initially have a very limited view of the object, which will improve as it moves closer to the object. On the other hand, static external cameras will always have the same view of the object. Additionally, there is a possibility that as the robot approaches the object, it may be occluded by other objects in the scene. In such cases, the robot is expected to utilize its motion to obtain a better view of the object.

Existing real-world object pose estimation benchmark datasets, such as the YCB Video dataset (Xiang et al., 2017), primarily consist of short single-camera video sequences without significant changes in the object’s view. The camera is positioned very close to the objects, simulating scenarios where the robot is already in close proximity to the object for grasping (i.e., assuming sequential navigation and manipulation). Furthermore, in multi-view datasets such as T-LESS (Hodaň et al., 2017), the object appears at an equal distance across all the views. Consequently, these datasets are not well-suited for evaluating this research. To address this gap, we contribute a “multi-view YCB object pose tracking dataset for mobile manipulation” (MY-MM).

##### 3.1.2.1 MY-MM dataset

**FIGURE 4:**
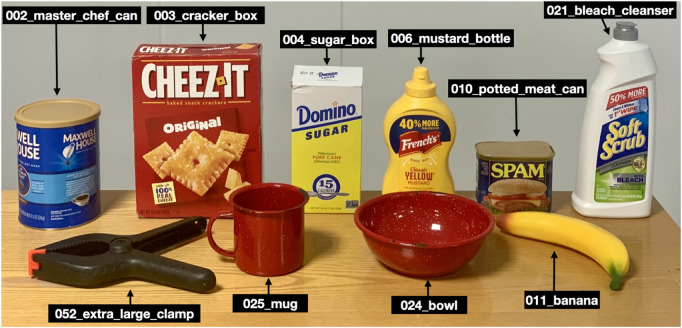
Subset of ten YCB objects used in the MY-MM dataset.

**FIGURE 5:**
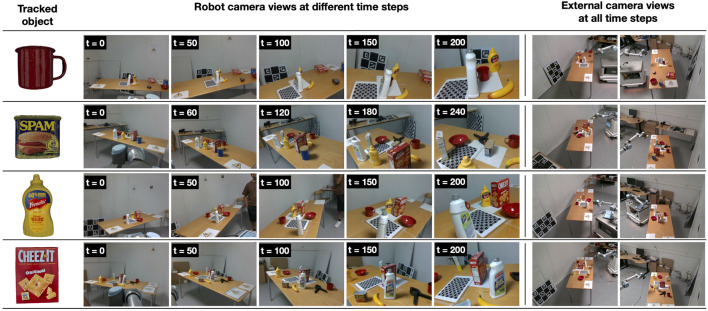
External and robot camera views at different time steps for four different sequences in the MY-MM dataset.

The dataset comprises 100 sequences featuring ten different YCB objects with distinct geometries and textures (Figure 4). For each video sequence (Figure 5), we provide the 6D pose of the object of interest in the robot camera frame, views of the scene from two external cameras, the transformations between all cameras for each time step, and intrinsic calibration for all the cameras. For each object, we provide ten sequences with ground truth poses. In each scene, along with the object with ground truth poses, several distractor objects are also present (Figure 5). Full robot camera videos along with the recorded ground truths for the sequences in Figure 5 can be viewed here^2^.

The external cameras are positioned approximately 3 m from the objects (Figure 1). The robot camera moves toward the object, starting from 2.5 m away and approaching to approximately 0.5 m from the object, recording approximately 250 frames in the process. The robot camera views were recorded using the Intel RealSense D435 RGB-D camera at a resolution of 640 × 480. External camera views were captured using Intel RealSense D455 RGB-D cameras at full HD resolution. However, only the center crop of the captured image, sized 640 × 480, containing a view of the table has been included in the dataset. We thus also reduce possible calibration errors caused by fish-eye effects in the periphery of the images.

##### 3.1.2.2 Ground truth annotation

**FIGURE 6:**
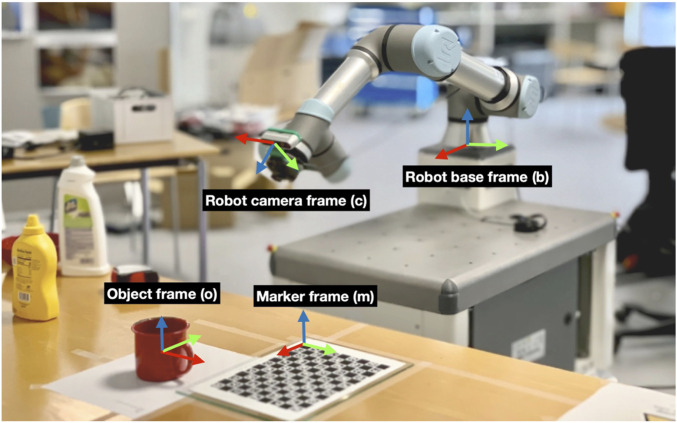
Setup for initially recording the transformation between the object and the robot TCP.

Computing the ground truth 6D pose from a distance is challenging. We employed the setup depicted in Figure 6 to mimic the robot camera views while approaching the object for grasping and to annotate the object’s ground truth pose. First, the relative transformation between the YCB object origin frame o and a marker origin frame m was manually measured. To minimize the errors, markers were printed with object outlines, and the objects were carefully placed inside. This was followed by estimating the marker pose in the robot camera frame c to estimate the relative transformation between these two frames. Since the transformations between the object frame o and marker frame m and a robot camera frame c and robot base frame b are already known, they can be used to compute object poses in the robot base frame r . Moreover, the transformation between the robot TCP frame and the robot camera frame is also known. The estimated object ground truth is then manually validated by overlaying the object CAD model on the image using the estimated ground truth.

**FIGURE 7:**
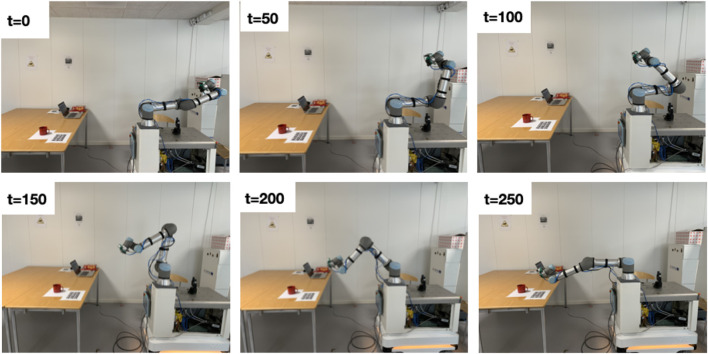
Simulating robot camera views while navigating toward the target object. Ground truth object pose at each time step is annotated by using the robot’s TCP pose at each time step and initially recorded transformation between the object and the robot’s TCP (Figure 6).

Each sequence is recorded as follows. Initially, the robot camera is positioned at a considerable distance from the object (Figure 7) at t=0 . At each subsequent time-step, the robot camera is moved closer to the object using only the manipulator motion. To ensure an accurate estimation of the object pose, the manipulator is completely stopped during each step to avoid any synchronization issues that may arise due to the motion. The transformation from the robot TCP to the robot base is recorded at each time-step. This recorded transformation and the pre-computed transformation from the robot base to the object are used to estimate the ground truth object pose. By following this procedure, the object pose with respect to the robot camera is progressively estimated as the camera approaches the object.

External camera views are obtained using cameras mounted on the wall (Figure 1). The extrinsic calibration of all the cameras, both robot and external, is initially performed with respect to a ChArUco marker.

#### 3.1.3 Evaluation metrics

We evaluate pose estimation performance using ADD and ADD-S metrics as previously proposed (Hinterstoisser et al., 2013; Xiang et al., 2017; Deng et al., 2021). Given the ground truth rotation R and translation T , the estimated rotation $\hat{R}$ and translation $\hat{T}$ , the set of 3D model points Y and the number of points |Y| , we define the following.

- *ADD:* The average distance computes the mean of the pairwise distances between the 3D model points transformed according to the ground truth and estimated pose as described in Equation 12.
$$\text{ADD}=\frac{1}{\left|Y\right|}\underset{x\in Y}{\sum }\|\left(Rx+T\right)-\left(\hat{R}x+\hat{T}\right)\|$$ (12)
- *ADD-S:* For symmetric and partially symmetric objects such as a bowl, mug, and banana, the match between points is ambiguous for some views. Therefore, the average distance is computed using the closest point distance as described in Equation 13.
$$\text{ADD}-\text{S}=\frac{1}{\left|Y\right|}\underset{x_{1}\in Y}{\sum }\underset{x_{2}\in Y}{\min}\|\left(Rx_{1}+T\right)-\left(\hat{R}x_{2}+\hat{T}\right)\|$$ (13)

The 6D pose is considered correct if the average distance is smaller than a pre-defined threshold. We report area under the curve (AUC) of ADD and ADD-S (Labbé et al., 2020) for threshold ranging from 0 to 10 cm in steps of 1 cm.

To evaluate the object pose uncertainty quantification, we use the following metrics.

- *Log probability of ground truth translation:* the log of the probability assigned to the ground truth translation in the estimate of the posterior translation distribution^3^ $P\left(T_{t}|Z_{1:t}\right)$ .
- *Log probability of ground truth rotation:* the log of the probability assigned to the ground truth in the estimate of the posterior rotation distribution $P\left(R_{t}|Z_{1:t}\right)$ .

These metrics assess whether the distribution accurately assigns probability mass to the ground truth translation and rotation. As the log is undefined for zero probability, the minimum probability values were clipped to 1e−5. Thus, the minimum value for the log probability of ground truth translation and ground truth rotation is $\log_{2}\left(1e-5\right)=-16.6$ .

#### 3.1.4 Hyperparameters and implementation details

We used a three-camera setup (two external cameras and one robot: N=3 ) for evaluation (Figure 1). As noted in Deng et al. (2021), approximately 100 particles result in optimal performance. Hence, during initialization, 40 particles were sampled around object detection in each camera frame. Thus, the total number of particles M was set to 120.

To initialize the particles, the translation estimate $\mu_{n,0}$ was determined using the center of the 2D bounding box of the object obtained using the Detectron2 (Wu et al., 2019) object detector^4^ and the corresponding depth in the depth image.

The particles were then sampled around these estimates, assuming normal distributed noise with a standard deviation $\Sigma_{n,t}$ of 20 pixels in the x and y estimates and 10 cm in the z estimate (Equation 6). The same strategy was also employed to initialize the particles for the baseline. The standard deviations $\Sigma_{n,t}$ can be increased if more noise is expected in the translation estimates used for initialization. However, the larger the noise, the more particles are required to uniformly cover the translation space.

During the multi-view fusion, $\gamma_{\text{thres}}$ was set to 0.6, as it was observed that pose distributions in the particular camera frame were unreliable when $\gamma_{\text{thres}}$ fell below 0.65 (Deng et al., 2021). $\epsilon_{\text{fus}}$ was set to 1e−5. $e_{\text{fus}}$ is used for additive filtering and hence should be a small value. For computing observation likelihoods, de-noising auto-encoders were trained for each object as detailed in Deng et al. (2021). To identify supporting samples, $\epsilon_{\text{sup}}$ was set to 30° and $\epsilon_{\text{min}}$ was set to 1e−5. $e_{\text{sup}}$ and $e_{\text{min}}$ were used for associating the rotational hypothesis from the previous time-step t−1 to rotation distribution at time t . Thus, for strict association, $e_{\text{sup}}$ should be decreased and $e_{\text{min}}$ increased. To decide whether the rotational hypothesis $r_{h,t}$ should be carried over to the next time-step, $c_{\text{thres}}$ was set to 0.1. The multiplicative inverse of $c_{\text{thres}}$ implicitly defines the maximum number of rotation hypotheses at a given time step. Therefore, the smaller the value of $c_{\text{thres}}$ , the larger the maximum number of rotation hypotheses that can be maintained. The unimodal or multi-modal nature of the posterior distributions was assessed using the Henze−Zirkler multivariate normality test (Henze and Zirkler, 1990).

All experiments were conducted on a workstation equipped with an Intel i9 processor, 32 GB RAM, and an NVIDIA GeForce RTX 3090 graphics card. The execution speed was approximately 4 Hz for the three-camera setup.

### 3.2 Results

In this section, we present the results of our experiments. We first provide a qualitative example in Section 3.2.1. Quantitative results that validate the objectives discussed in Section 3.1.1 are presented in Section 3.2.2.

#### 3.2.1 Qualitative results

**FIGURE 8:**
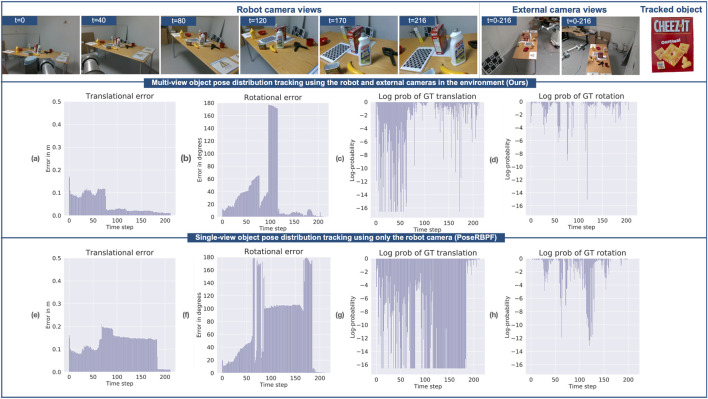
Comparison of object pose distribution tracking using Ours and PoseRBPF (Deng et al., 2021). First row: Robot views at different time frames, external camera views, tracked object (cracker box). Second row: **(a)** translational error, **(b)** rotational error, **(c)** log-probability of ground truth translation and **(d)** log-probability of ground truth rotation for ours. Third row: **(e)** translational error, **(f)** rotational error, **(g)** log-probability of ground truth translation and **(h)** log-probability of ground truth rotation for PoseRBPF.

Figure 8 demonstrates the role of external cameras in preventing the particle filter from diverging, even when the object becomes severely occluded in the robot camera view. Initially (t=0−30) , and the object is visible in the robot camera view. However, translation errors in the estimated object pose are notably high when using both the robot and external cameras (Figure 8a) and when using only the robot camera (Figure 8e). This is attributed to noisy depth images due to the considerable distance (approximately 3 m) between the camera and the object. However, rotational errors remain low in both scenarios (Figures 8b,f), since a unique view of the object is available in the robot camera during this period.

As the robot approaches the object, it becomes increasingly occluded in the robot camera view (t=30−120) , resulting in an increase in errors in both scenarios. However, there is a noteworthy difference in how these errors evolve with and without the use of external cameras. In the case of multi-view (Figure 8b), the rotational error increases as the filter starts to rely on observations from external cameras which are more uncertain than robot camera views without occlusion. However, evidence provided by the external cameras during this period prevents the particle filter from diverging (Figure 8c), and as a result, it quickly converges once the object starts to re-appear in the robot camera view. Quick convergence and accurate quantification of uncertainty can enable the robot to make early and reliable decisions as it approaches the object for grasping. Conversely, while just using a robot camera, the filter starts to diverge (Figure 8g) due to noisy evidence provided by the robot camera during this period. As a result, there is a large increase in translational (Figure 8e) and rotational (Figure 8f) errors. Eventually, the filter converges at the end of the sequence (Figures 8e,f).

More qualitative results can be found here^5^.

#### 3.2.2 Quantitative results

Table 1 presents pose estimation results using two baselines—PoseRBPF (Deng et al., 2021) and CosyPose (Labbé et al., 2020) —and the proposed framework. As PoseRBPF is a single-view pose estimation method, we present pose estimates in a robot and two external cameras. These single-view pose estimates were then used as an input to CosyPose for refinement^6^. For large objects with distinct features, external cameras result in better performance as they have an unoccluded view of the object as well as sufficient pixel information for pose estimation. The performance also depends on the view of the object, as the external cameras are stationary.

**TABLE 1:** Pose estimation results.

	AUC of ADD					AUC of ADD-S				
	PoseRBPF			CosyPose	Ours	PoseRBPF			CosyPose	Ours
Object	Robot cam	External camera 1	External camera 2	Robot + external cameras	Robot + external cameras	Robot camera	External camera 1	External camera 2	Robot + external cameras	Robot + external cameras
002_master_chef_can	*22.48*	40.73	20.10	24.94	**46.93**	*69.68*	**85.02**	81.59	77.91	84.48
003_cracker_box	59.22	**65.07**	*19.79*	65.04	62.29	79.00	**87.20**	*72.94*	82.77	82.76
004_sugar_box	45.76	08.30	*21.05*	36.88	**47.69**	64.32	58.22	*39.54*	52.67	**77.55**
006_mustard_bottle	43.42	*29.78*	50.27	**48.59**	43.04	74.56	**85.05**	*67.04*	75.02	80.84
010_potted_meat_can	*09.22*	28.34	19.39	10.96	**33.13**	*24.53*	**65.28**	36.82	25.59	60.97
011_banana	30.11	*05.64*	20.15	**43.20**	22.46	50.87	*18.98*	42.18	**60.99**	40.30
021_bleach_cleanser	39.55	09.82	*04.69*	39.63	**55.73**	64.85	46.46	*33.94*	60.05	**77.51**
024_bowl	19.16	23.68	*24.59*	25.14	**37.46**	*35.17*	62.86	**65.27**	39.73	64.53
025_mug	*20.25*	49.54	33.18	26.96	**64.77**	*53.75*	80.96	83.50	54.04	**87.11**
052_extra_large_clamp	34.35	11.42	*10.61*	**37.91**	27.35	53.04	28.54	*37.40*	**55.03**	48.57
All	32.35	27.23	*22.38*	35.92	**44.08**	56.97	61.85	*56.02*	58.38	**70.46**

For colored objects ADD-S, metric is more relevant due their symmetry; for other objects ADD, metric is more relevant.

Bold indicates the best performing method.

CosyPose is able to slightly improve the pose estimates obtained using PoseRBPF in the robot camera by refining them using the pose estimates obtained using PoseRBPF in the external cameras. However, the difference is not significant, since the estimates obtained in the external cameras have large errors, especially for small objects.

Our framework achieves significantly better performance on objects such as the “006_mustard_bottle,” “010_potted_meat_can,” “021_bleach_cleanser,” “024_bowl,” and “025_mug.” This is because these objects are partially symmetric and have smaller sizes. As a result, during occlusion in the robot camera, they are either very severely occluded or have ambiguous views. The additional views from external cameras prevent the particle filter from diverging during occlusions when the robot camera has a poor view of the object. As a result, the particle filter quickly converges to the accurate pose estimate when the object once again has a good view in the robot camera. Without the additional views from the external cameras, the particle filter often needs to be re-initialized after occlusions (Deng et al., 2021). In the case of objects like “011_banana” and “052_extra_large_clamp,” the robot and external cameras often have contradictory observations that cannot be reliably captured by the failure model (Section 2.3.4); this leads to inaccurate distributions after fusion.

In Table 2, we present pose uncertainty quantification results using PoseRBPF (Deng et al., 2021) and the proposed framework. The proposed framework results in more accurate translation distributions, as indicated by the high log probability of ground truth translation. This is because, when sufficient evidence is not available, it prevents premature convergence and maintains multimodal translation distributions, while PoseRBPF incorrectly converges to inaccurate translation distributions. Preventing premature convergence is crucial for preventing the particle filter from diverging when the robot camera has poor views of the object.

**TABLE 2:** Pose uncertainty quantification results.

Object	Log probability of ground truth translation		Log probability of ground truth rotation	
	PoseRBPF	Ours	PoseRBPF	Ours
	Robot camera	Robot + external cameras	Robot camera	Robot + external cameras
002_master_chef_can	*−3.05*	**−0.46**	***−5.88***	−5.98
003_cracker_box	−3.06	**−1.56**	−3.04	**−2.98**
004_sugar_box	−4.37	**−2.04**	**−6.04**	−6.35
006_mustard_bottle	−3.53	**−1.95**	−4.23	**−3.63**
010_potted_meat_can	*−10.57*	**−4.18**	*−8.63*	**−6.54**
011_banana	−5.08	**−4.70**	−5.49	**−4.27**
021_bleach_cleanser	−4.90	**−2.01**	−5.41	**−4.82**
024_bowl	−5.42	**−0.97**	*−4.42*	**−3.96**
025_mug	*−5.46*	**−0.31**	*−7.33*	**−4.35**
052_extra_large_clamp	−3.03	**−0.95**	−7.19	**−5.81**
All	−5.02	**−1.91**	−5.76	**−4.86**

Bold indicates the best performing method.

Additionally, in the case of objects with distinct features and larger sizes such as the “003_cracker_box,” PoseRBPF has a slightly higher probability mass in the ground truth rotation. This is because if a robot camera has a good view of the object and a well-estimated translation, external cameras due to their large distance to the object can introduce noise during fusion. However, the use of external cameras results in much better rotation distributions for other objects that are partially symmetric and have smaller sizes.

#### 3.2.3 Ablation studies

In this section, we first present three ablation studies to validate our design choices, followed by an ablation study on pose estimation and uncertainty quantification using different camera combinations for fusion.

##### 3.2.3.1 Multi-view fusion

In this study, we validate the fusion approach outlined in Section 2.3.4. We compare our approach against two baselines. In the first, we directly fuse all the observations (similar to Naik et al. (2022)), while in the second baseline we perform additive filtering before fusion. Our approach (“Ours”), in addition to additive filtering, uses a failure model described in Section 2.3.4 to determine whether a particular observation should be included during the fusion. In the experiments, all particles at each time-step were injected using ground truth translations to ensure that design choices during the other steps in the particle filter did not affect the fusion. We measure the performance using the log of probability assigned to the ground truth rotation in the fused distribution. Results are presented in Table 3.

**TABLE 3:** Ablation study on multi-view fusion.

Object	Log probability of ground truth rotation		
	Fusion without additional filtering	Fusion with additional filtering	Ours (Section 2.3.4)
002_master_chef_can	−14.26	−7.32	**−7.08**
003_cracker_box	−14.74	−5.45	**−4.74**
004_sugar_box	−16.53	−8.06	**−5.48**
006_mustard_bottle	−13.34	−6.05	**−3.46**
010_potted_meat_can	−16.41	−6.17	**−3.59**
011_banana	−16.60	−5.61	**−3.70**
021_bleach_cleanser	−16.60	−7.25	**−4.03**
024_bowl	−15.70	−7.13	**−6.93**
025_mug	−15.05	−5.60	**−4.93**
052_extra_large_clamp	−16.60	−7.71	**−6.07**
All	−15.52	−6.63	**−5.00**

Bold indicates the best performing method.

Fusion without additive filtering results in poor performance (Table 3) because if the object is not visible in one of the cameras, observations from other cameras are also lost. Fusion with additive filtering addresses this and significantly improves performance as it retains good observations from other cameras before fusion. However, it does not effectively handle situations in which one of the cameras provides a poor view of the object, resulting in noisy observations. Fusing such noisy observations with additive filtering has the potential to corrupt good observations from other cameras. To mitigate this, we employ the failure model described in Section 2.3.4 to determine whether a particular observation should be included during fusion, which results in improved performance—as indicated by the high log probability of ground truth rotation in the right column of Table 3.

##### 3.2.3.2 Re-sampling

In this study, we validate the re-sampling approach outlined in Section 2.3.5. We compare our approach against two baselines. In the first, we re-sample all particles simultaneously using low-variance re-sampling (Thrun, 2002; Deng et al., 2021; Naik et al., 2022). In the second baseline, we separately re-sample particles originating from different camera observations at t=0 , so that particles from all camera observations are retained throughout tracking. Our approach (“Ours”) dynamically adjusts the number of particles originating from different camera observations at t=0 based on the particles’ aggregate weight. Subsequently, these particles are separately re-sampled, as described in Section 2.3.5. The performance of all three approaches is measured by comparing the log probability of the ground truth translation in the resultant translation distribution.

Re-sampling all the particles simultaneously (first baseline) leads to improved performance for large objects with distinct features, as demonstrated in Table 4, compared to our approach and the second baseline. This is because the robot camera often provides high-quality observations of these objects since they are rarely fully occluded due to their large size, while external cameras tend to have comparatively noisy observations due to their greater distance from the object. For these objects, simultaneously re-sampling all particles quickly eliminates noisy particles from the external cameras, resulting in more compact translation distributions than our approach, where particles from all cameras are retained until sufficient temporal evidence is available or the second baseline, where particles originating from different frames are retained at all times.

**TABLE 4:** Ablation study on re-sampling.

Object	Log probability of ground truth translation		
	Re-sampling all particles simultaneously	Separately re-sampling particles originating from different frames	Ours (Section 2.3.5)
002_master_chef_can	**−0.80**	−1.35	−1.67
003_cracker_box	**−2.08**	−2.71	−2.63
004_sugar_box	**−2.81**	−4.60	−3.74
006_mustard_bottle	**−1.32**	−2.38	−2.62
010_potted_meat_can	−6.67	−6.13	**−0.42**
011_banana	−5.10	−6.92	**−3.40**
021_bleach_cleanser	−5.79	−6.47	**−2.68**
024_bowl	−1.30	−1.16	**−0.54**
025_mug	−3.91	−1.94	**−0.52**
052_extra_large_clamp	−2.92	−5.11	**−2.18**
All	−3.27	−3.87	**−2.04**

Bold indicates the best performing method.

However, the advantage of our approach is clearly evident in the case of challenging objects where the robot camera does not always provide a good observation due to partial symmetry or smaller object sizes. Our approach enables particles from different cameras to refine their estimates instead of quickly converging to specific estimates, as observed in the first baseline. Furthermore, it does not retain noisy particles all the time, as in the second baseline. Overall, then, our approach outperforms both baselines, particularly in the case of challenging objects (Table 4).

##### 3.2.3.3 Pose estimation

In this study, we validate the pose estimation from the tracked distribution. We use the approach in Deng et al. (2021) and Naik et al. (2022) as the baseline, where the pose is estimated using the mean of the samples in the current distribution that could be associated with the pose estimate at the previous time-step t−1 . Our approach (“Ours”), in addition to the samples in the current distribution that could be associated with the pose estimate at the previous time-step t−1 , also considers the samples that could not be associated. As a result, it maintains a multiple set of hypotheses when sufficient evidence is not available to converge to a single pose estimate (Section 2.3.6).

The same tracked distribution was used to evaluate both methods, with performance measured using the AUC of ADD and ADD-S metrics.

It can be observed from Table 5 that Deng et al. (2021) generally perform poorly, as they assume that a good initial object pose estimate is available. This assumption is often not valid when tracking from a distance. Our approach maintains a multiple pose hypothesis when sufficient temporal evidence is not then available t in support of the then pose estimate t−1 . It is thus able to recover from poor pose estimates and hence results in better performance.

**TABLE 5:** Ablation study on pose estimation from tracked distribution.

Object	AUC of ADD		AUC of ADD-S	
	*PoseRBPF*	Ours (Section 2.3.6)	*PoseRBPF*	Ours (Section 2.3.6)
002_master_chef_can	27.65	**29.19**	**82.11**	80.38
003_cracker_box	41.67	**55.80**	74.23	**81.14**
004_sugar_box	35.09	**42.95**	71.40	**73.67**
006_mustard_bottle	47.79	**50.01**	78.71	**82.88**
010_potted_meat_can	29.32	**35.66**	**74.00**	73.19
011_banana	19.39	**32.03**	52.97	**54.40**
021_bleach_cleanser	35.72	**49.67**	71.41	**76.83**
024_bowl	25.12	**37.63**	70.86	**74.03**
025_mug	42.18	**62.18**	86.66	**86.69**
052_extra_large_clamp	09.81	**11.34**	35.10	**35.54**
All	31.37	**40.64**	69.74	**71.87**

Bold indicates the best performing method.

#### 3.2.4 Different camera combinations

In this ablation study, we compare the performance of pose estimation and uncertainty quantification with fusion using different camera combinations. We consider three cases: i) robot camera and external camera 1 (EI); ii) robot camera and external camera 2 (E2); iii) robot camera, E1, and E2 (refer to Figure 1 for the positioning of the cameras). Results are presented in Table 6. The combination of the robot camera and E1 yields better performance than the combination of the robot camera and E2. The performance using the combination of the robot camera and E2 is very similar to the performance of just using the robot camera (Table 1). This is because E1 provides more informative views as it is positioned at a lower elevation than E2 (relative to the object frame). Consequently, E1 frequently has better views of the object’s front, back, and sides, which typically contain more unique features. In contrast, E2, due to its higher elevation, often has a better view of the object’s top compared to the views of its sides, front, and back.

**TABLE 6:** Different camera combinations for fusion.

Object	Pose estimation						Uncertainty quantification					
	AUC of ADD			AUC of ADD-S			Log probability of ground truth translation			Log probability of ground truth rotation		
	R-E1	R-E2	R-E1-E2	R-E1	R-E2	R-E1-E2	R-E1	R-E2	R-E1-E2	R-E1	R-E2	R-E1-E2
002_master_chef_can	*47.61*	25.74	***46.93***	83.93	77.49	**84.48**	−0.79	−1.17	**−0.46**	−6.03	**−5.57**	−5.98
003_cracker_box	55.57	47.19	**62.29**	**80.32**	79.63	**82.76**	−2.10	−2.18	**−1.56**	−3.46	−3.44	**−2.98**
004_sugar_box	38.98	42.76	**47.69**	**77.89**	70.95	77.55	**−1.58**	−4.71	−2.04	**−6.11**	−7.84	−6.35
006_mustard_bottle	35.03	**46.23**	43.04	**83.52**	77.06	80.84	**−1.26**	−3.11	−1.95	−4.11	**−3.48**	−3.63
010_potted_meat_can	*27.02*	08.05	***33.13***	47.90	19.28	**60.97**	−6.42	−9.85	**−4.18**	−6.93	−7.17	**−6.54**
011_banana	12.92	**27.90**	22.46	25.46	**47.39**	40.30	−6.18	**−3.64**	−4.70	−4.67	**−3.46**	−4.27
021_bleach_cleanser	50.68	47.44	**55.73**	73.26	63.61	**77.51**	−3.19	−5.00	**−2.01**	−5.11	−5.04	**−4.82**
024_bowl	34.56	26.79	***37.46***	61.69	46.65	**64.53**	−1.42	−3.84	**−0.97**	−3.99	**−3.87**	−3.96
025_mug	*59.22*	18.96	***64.77***	79.08	37.96	**87.11**	−1.25	−6.84	**−0.31**	**−3.70**	−6.66	−4.35
052_extra_large_clamp	**28.28**	23.62	27.35	46.50	39.51	**48.57**	−3.05	−5.06	**−0.95**	−7.16	−7.85	**−5.81**
All	38.98	31.46	**44.08**	65.95	55.95	**70.46**	−2.72	−4.54	**−1.91**	−5.12	−5.43	**−4.86**

R, robot camera; E1, external camera 1; E2, external camera 2.

Bold indicates the best performing method.

However, when all the cameras are used together, there is a significant improvement in performance compared to using only the robot camera and E1. This is because E1 and E2 provide complementary views of the object (both are placed on opposite sides of the object; see Figure 1). These complementary views help resolve uncertainties and lead to improved pose estimates and uncertainty quantification.

### 3.3 Application: pre-grasp planning

We applied the proposed multi-view object pose distribution tracking framework for making informed decisions at various stages of mobile manipulation (MM). As described in Section 1, in addition to selecting the optimal grasp pose for grasping the object (Newbury et al., 2022), MM also requires selecting the optimal base pose from where the object can be grasped (Vahrenkamp et al., 2013; Makhal and Goins, 2018; Jauhri et al., 2022b).

An estimate of object pose $\hat{X}$ is required to select the optimal base poses and grasp poses for grasping the objects. Additionally, estimates of the selected optimal grasp poses and base poses are also derived from the estimated object pose. Therefore, any error in estimated object pose $\hat{X}$ can result in failures. An error in the base pose can potentially result in a situation where there are no inverse kinematics (IK) solutions for grasping the object after navigating to the estimated base pose, while an error in the estimated grasp pose might lead to a failed grasp.

As the proposed framework estimates the object pose $\hat{X}$ and the associated uncertainty P(X) from a distance, the robot can make early decisions regarding the optimal base pose and grasp pose for grasping the object. Moreover, instead of directly performing the actions—navigating to the estimated base pose or attempting to grasp using the estimated grasp pose—the robot can project the object pose uncertainty P(X) onto the action space and assess whether the estimated uncertainty is acceptable for the successful execution of the action.

For the base pose, the robot can evaluate if deviations according to the uncertainty associated with the estimate of the selected base pose would still allow for IK solutions for grasping the object. Similarly, for the grasp pose, it can validate in the simulation (Kim et al., 2012) whether the deviations according to the uncertainty associated to the estimate of the selected optimal grasp pose would enable a successful grasp, as a gripper can compensate for the pose uncertainty. For mathematical details, we refer the reader to Naik et al. (2025).

In our experiments, we assumed that the top-down grasp pose was selected as the optimal grasp pose for grasping the objects. We examined four cases, each involving the use of P(X) for decision-making at an increased number of stages than the previous case.

- Fixed base pose and object pose estimation (FBOE): the robot goes to a fixed base pose near the table, estimates object pose, and attempts to grasp the object using the estimate of the selected grasp pose. This represents how MM is usually solved.
- Object pose tracking and base pose estimation (OTBE): the robot starts tracking the object pose by means of our method, obtains an estimate of the selected base pose, and navigates to the estimated base pose. It then obtains the estimate of the selected grasp pose and attempts to grasp the object.
- Object pose tracking and base IK monitoring (OTBM): OTBE plus the robot monitors the probability of the availability IK solution using the estimate of the selected base pose and the uncertainty in the object pose used for estimating the base pose. The robot decides to navigate to the estimated base pose only if the probability of success exceeds a pre-defined threshold.
- Object pose tracking and base IK and grasp success monitoring (OTBGM): OTBM plus the robot monitors the probability of grasp success using the estimate of the selected grasp pose and the uncertainty in the object pose used for estimating the grasp pose. The robot decides to attempt to grasp using the estimated grasp pose only if the probability of success exceeds a pre-defined threshold.

In OTBM and OTBGM, the pre-defined threshold for both base and grasp poses was set to 60%—that is, if more than 60% of the errors could be compensated for, the action was executed. In practice, aside from errors in visual object pose estimates, several other factors can contribute to failures during action execution. These may include poor calibration between the external camera and the robot camera due to errors in robot localization, and inability to plan a trajectory to the intended grasp pose due to occlusion from other objects. To isolate these failures and only quantify failures due to errors in the visual object pose estimate, we created digital twins for the sequences in the MY-MM dataset using the recorded ground truths and NVIDIA Isaac simulator^7^. The decisions were based on the real-world data from the MY-MM dataset sequences, while actions were executed in the digital twin.

We evaluated five objects (“003_cracker_box,” “006_mustard_bottle,” “021_bleach_cleanser,” “024_bowl,” and “025_mug”) with diverse shapes, colors, and textures, conducting ten trials for each object and each case. FBOE and OTBE do not consider uncertainty in the object pose, resulting in a large number of failures (Table 7).

**TABLE 7:** Grasp success rate with and without using visual object pose uncertainty during decision-making.

Object	FBOE		OTBE		OTBM		OTBGM	
	SG	AG	SG	AG	SG	AG	SG	AG
003_cracker_box	8	10	7	10	10	10	10	10
006_mustard_bottle	1	10	3	10	5	10	4	5
021_bleach_cleanser	3	10	3	10	5	8	4	7
024_bowl	4	10	3	10	7	9	7	9
025_mug	6	10	4	10	10	10	10	10
All	22	50	20	50	37	47	**35**	41
Success rate	44%		40%		78.72%		**85.36%**	

SG, successful grasps; AG, attempted grasps.

Bold indicates the best performing method.

On the other hand, OTBM and OTBGM monitor uncertainties in the tracked object pose before making MM decisions. Consequently, they exhibit a much higher success rate and fewer failures. Most of the failures in OTBGM occurred due to the absence of a valid IK solution at the estimated base pose. To mitigate these failures, one option is to raise the threshold for decision-making, requiring more than 60% of estimated errors to be compensated for before executing the action. However, this strategy comes with the trade-off of delaying decision-making, which results in longer execution times.

In summary, the early pose estimates from the proposed object pose distribution tracking framework not only empower the mobile robot to make timely decisions regarding the optimal base pose and grasp pose for object manipulation but also the associated pose uncertainty, provided by the proposed framework, helping to prevent failures. In this way, the proposed framework contributes to advances in both the *time efficiency* and *robustness* of mobile manipulation.

## 4 Discussion

In this study, we have presented a particle filter-based framework for multi-view 6D object pose distribution tracking using the robot’s onboard camera and external cameras in the environment. We demonstrated that the proposed framework yields more accurate 6D object pose estimates than existing multi- and single-camera pose estimation methods and distributions compared to existing single-camera pose distribution estimation methods as the mobile robot approaches the objects for grasping. The external cameras not only help resolve ambiguities when the robot camera has ambiguous views of the object but also prevent the particle filter from diverging. Consequently, the particle filter quickly converges once the robot camera views start to improve, unlike when using only the robot camera, where the particle filter needs to be re-initialized. Finally, we contributed a *multi-view YCB object pose tracking dataset for mobile manipulation* that can serve as a benchmark for future research in this direction.

Our results are encouraging, as they indicate that the use of external cameras can significantly improve the accuracy of the estimated object pose and distributions in the robot camera. This can enable the mobile robot to make early and informed decisions, thereby improving both the *time efficiency* and *robustness* of mobile manipulation.

We here used the PoseRBPF method (Deng et al., 2021) for computing a discretized rotational distribution, which uses Euler angle representation. However, this introduces a risk of Gimbal lock during multi-view fusion. Future research should investigate the use of quaternions for discretizing SO(3), as proposed by Murphy et al. (2021) and Haugaard et al. (2023). Furthermore, the single-view observation likelihood model from PoseRBPF (Deng et al., 2021) used in this work exhibits reduced performance on YCB objects such as “011 banana” and “052 extra-large clamp” under occlusions. This highlights the need for more robust single-view observation likelihood models capable of handling such challenging cases. Finally, our framework can be combined with a partially observable Markov decision framework (PO-MDP), as applied in An et al. (2024), to determine actions that will reduce uncertainty and provide better pose estimates and distributions.

## Data Availability

The datasets presented in this study can be found in online repositories. The names of the repository/repositories and accession number(s) can be found at: https://nextcloud.sdu.dk/index.php/s/eQitCanx9ztLZwg.

## Footnotes

1 It should be noted that the observation likelihood $P\left({\;\;}^{n}\;Z_{t}|T_{t}^{m},R_{t}^{m}\right)$ is computed for a specific observation Ztn for a set of poses sampled on SE(3). Consequently, the histogram expressing the observation likelihood is not a normalized probability distribution. The likelihood of each bin is calculated using a cosine distance which has a value in the range of 0–1.

2
https://lakshadeep.github.io/mymm/.

3 It should be noted that in the case of continuous distributions such as a mixture of Gaussians used for modeling $P\left(T_{t}|Z_{1:t}\right)$ , the probability assigned to the ground truth translation is actually a likelihood.

4 It was found that the Detectron2 models trained on the photo-realistic data for detecting the objects in the external cameras were very sensitive to the seeds. Hence, four Detectron2 models were trained with different initial weights. For each object class, validation data were used to determine the best-performing Detectron2 model. For reproducibility and comparative evaluation with future research, we have made these detections available in the dataset.

5
https://lakshadeep.github.io/mvopdt/.

6 The single-view pose estimator proposed in CosyPose performed poorly on the MY-MM dataset when the distance between the objects and the camera was large. We attribute this to the fact that the pre-trained models were trained on data where the objects were typically close to the camera and did not use depth information. Therefore, for a fair comparison, we used poses estimated using PoseRBPF in each camera at all time steps and refined them using the multi-view refinement method proposed in CosyPose.

7
https://developer.nvidia.com/isaac-sim.
